# High-flow nasal cannula oxygen therapy versus noninvasive ventilation versus in immunocompromised patients with acute respiratory failure

**DOI:** 10.1186/2197-425X-3-S1-A98

**Published:** 2015-10-01

**Authors:** R Coudroy, JP Frat, P Petua, R Robert, A Jamet, AW Thille

**Affiliations:** CHU Poitiers, Service de Réanimation Médicale, Poitiers, France; INSERM CIC 1402 (Equipe 5 ALIVE), Université de Poitiers, Poitiers, France

## Introduction

Acute respiratory failure (ARF) is the leading cause of intensive care unit (ICU) admission of immunocompromised patients. In the early 2000's, two randomized controlled trials suggested that noninvasive ventilation (NIV) could decrease mortality of immunocompromised patients admitted to ICU for acute respiratory failure (ARF) as compared to standard oxygen therapy (O_2_). However, the benefits of NIV in immunocompetent patients with ARF are debated. High flow nasal cannula oxygen therapy (High-Flow Oxygen) may offer an alternative in hypoxemic patients and we recently found in a randomized controlled trial including 310 patients with ARF that High-Flow Oxygen decreased mortality as compared to NIV [1]. In our unit, High-Flow Oxygen is increasingly used in our unit gradually replacing the use of NIV.

## Objectives

To compare outcomes of immunocompromised patients admitted to ICU for ARF and treated with High-Flow Oxygen or NIV as first-line therapy.

## Methods

All patients admitted for ARF to our 15-bed medical ICU of a teaching hospital from January, 1^st^ 2007 to December, 31^th^ 2014 were retrospectively screened. Among them we included those who were immunocompromised including haematological or solid cancer, neutropenia or immunosuppressive therapy such as corticosteroids or oral chemotherapy, and who received High-Flow Oxygen or NIV as first-line therapy. Patients with acute-on-chronic respiratory failure, those treated with standard oxygen alone or needing immediate intubation, and those with a do-no-intubate order were excluded.

## Results

Over this 8-year period, 1299 patients were admitted for ARF including 267 (21%) immunocompromised patients. After excluding 142 patients, 125 patients were analyzed including 52 patients (42%) treated with High-Flow Oxygen and 73 patients (58%) with NIV. Baseline characteristics were not different between the 2 groups. Patients were more likely to be treated with NIV in the first period study (2007-2010): 77% versus 47% in the second period (2011-2014), p = 0.0015. Intubation rate was lower in patients treated with High-Flow Oxygen (33%) than in those treated with NIV (53%, p = 0.03). In-ICU mortality and day-28 mortality were significantly lower in patients treated with High-Flow Oxygen than in those treated with NIV (15% vs. 33%, p = 0.04; and 17% vs. 38%, respectively, p = 0.02). After adjustment on SAPS II, the use of NIV remained independently associated with day-28 mortality with an adjusted odds ratio of 3.04 [1.36-6.78], p = 0.005.

## Conclusions

In immunocompromised patients admitted to ICU for ARF, intubation and mortality rates were lower in patients treated with High-Flow Oxygen than in those treated with NIV as first-line therapy. The use of NIV was independently associated with day-28 mortality.
